# Neurotrophin NGF/TrkA and BDNF/TrkB signaling orchestrates the immune microenvironment in osteosarcoma

**DOI:** 10.3389/fimmu.2025.1727434

**Published:** 2026-01-06

**Authors:** Hongyuan Liu, Guobing Wang, Chunxue Wang, Dan Hou, Shigang Li, Yuansheng Fan, Bing Liu

**Affiliations:** 1 Orthopedics and Traumatology Department, Yibin Traditional Chinese Medicine Hospital, Yibin, China; 2 Medical Research Laboratory, Yibin Traditional Chinese Medicine Hospital, Yibin, China; 3 Outpatient, Yibin Traditional Chinese Medicine Hospital, Yibin, China

**Keywords:** antitumor immunity, dendritic cells, myeloid polarization, neurotrophins, osteosarcoma, tumor-associated macrophages

## Abstract

Neurotrophin signaling through NGF/TrkA and BDNF/TrkB is increasingly recognized as a driver of osteosarcoma (OS) progression and an organizer of its immune milieu, yet clinical translation has lagged amid intratumoral heterogeneity and a myeloid-skewed, vasculature-aberrant tumor microenvironment (TME). Features that blunt immune competence include dominant tumor-associated macrophage programs, sparse and dysfunctional effector T cells, endothelial remodeling that restricts lymphocyte entry, and neuron–immune circuits that reinforce suppression. Within this context, NGF/TrkA promotes matrix remodeling, monocyte ingress, and macrophage polarization, while BDNF/TrkB modulates dendritic-cell maturation, supports survival and angiogenesis, and may condition T-cell priming—together positioning neurotrophins as coordinators of tumor persistence and immune exclusion. This review surveys these mechanisms and maps them to therapeutic strategies: kinase-level blockade with approved TRK inhibitors in NTRK fusion–positive disease; exploratory pathway inhibition in fusion-negative OS; ligand-directed approaches; and rational combinations with immunotherapy and vascular/stromal modulators. We highlight biomarker frameworks (receptor–ligand activity scores, phospho-Trk immunohistochemistry, NGF–MMP-2 readouts) and safety considerations that should structure early-phase trials. Clinical and preclinical signals collectively support testing neurotrophin-targeted strategies to recalibrate myeloid composition, enhance antigen presentation, and restore T-cell access to tumor beds. The purpose of this review is to synthesize current evidence and propose a translational roadmap for targeting NGF/TrkA and BDNF/TrkB to remodel antitumor immunity in osteosarcoma.

## 1 Introduction

Osteosarcoma is a high-grade primary bone malignancy in which therapeutic progress has been limited by intratumoral heterogeneity and an immunosuppressive tumor microenvironment (1–3). Initially defined as regulators of neuronal development and nerve–tumor interactions, neural mediators including neurotrophins and their receptors are now recognized as drivers of tumor progression, host immunity and, via oncogenic Trk fusions, cancer therapy. Among these, nerve growth factor (NGF) binding to tropomyosin receptor kinase A (TrkA) and brain-derived neurotrophic factor (BDNF) binding to TrkB represent two conserved signaling axes with direct relevance to osteosarcoma biology and its immune milieu (4–6). Contemporary analyses of the osteosarcoma microenvironment underscore the abundance of myeloid populations, the scarcity and dysfunction of effector T cells, and stromal programs that collectively blunt antitumor immunity (7–9), framing a setting in which neurotrophin signaling could recalibrate leukocyte behavior and treatment responsiveness.

NGF/TrkA signaling is increasingly implicated in sarcoma biology. Transcriptomic interrogation of patient datasets shows NGF and TrkA expression enriched in osteosarcoma relative to other neurotrophins and receptors, with NGF associated with migratory and metastatic programs (10–12). Mechanistic experiments linked NGF–TrkA activity to matrix remodeling and proinvasive signaling, nominating this axis as a therapeutic vulnerability (13–15). These observations align with broader oncologic literature positioning NGF/TrkA as a driver of proliferation, survival, and motility through MAPK and PI3K–AKT cascades.

BDNF/TrkB signaling has been connected to malignant phenotypes across epithelial and neuroendocrine tumors and engages pathways that promote survival, invasion, and angiogenesis. In bone tumors, BDNF can induce VEGF and enhance endothelial interactions, suggesting a route by which TrkB may contribute to vascular remodeling in mineralized tissues (16–18). Beyond effects on cancer cells, TrkB is expressed by human dendritic cells, and BDNF alters their maturation, indicating plausible interfaces with antigen presentation and T-cell priming. These tumor-cell-intrinsic and immunomodulatory activities place the BDNF/TrkB axis as a candidate determinant of antitumor immune quality in osteosarcoma.

Neurotrophin–immune crosstalk is further supported by studies showing that NGF–TrkA signaling regulates leukocyte adhesion and trafficking programs and that nociceptive pathways—and their neurotrophin ligands—shape the inflammatory tone of the tumor bed. These data integrate with a broader framework in which nerve–cancer interactions remodel tissue compartments, influence stromal states, and modulate immune surveillance (19–22). In osteosarcoma, where perineural and axonogenic cues are increasingly recognized within the microenvironment, neurotrophin signaling likely participates in the coordination of myeloid polarization, lymphocyte exclusion, and endothelial activation that together constrain immune-mediated control.

This mini review synthesizes current knowledge on NGF/TrkA and BDNF/TrkB axes in osteosarcoma with emphasis on neurotrophin-driven remodeling of antitumor immunity. This mini review summarizes neurotrophin biology relevant to bone sarcoma, delineates mechanisms by which these pathways reprogram immune and stromal compartments, and evaluates therapeutic strategies ranging from ligand/receptor blockade to clinically available TRK inhibitors, with the objective of defining tractable approaches to restore immune competence in this disease.

## 2 NGF/TrkA and BDNF/TrkB biology in osteosarcoma

NGF and BDNF signal through the high-affinity tyrosine-kinase receptors TrkA and TrkB, respectively, which are expressed as multiple transcript and protein isoforms, with context-dependent cooperation or opposition from p75^NTR^ (23–25). Ligand engagement induces receptor autophosphorylation and adaptor recruitment, activating Ras–RAF–MEK–ERK, PI3K–AKT–mTOR and PLCγ cascades that couple survival, motility and cytoskeletal remodeling to shifts in glycolysis, oxidative phosphorylation and lipid metabolism, influencing immune-cell function, including T-cell exhaustion and myeloid suppressive metabolism. These canonical outputs, defined across multiple tumor types, provide a mechanistic scaffold for understanding osteosarcoma (OS) biology where neurotrophin cues intersect with lineage (mesenchymal) and microenvironmental constraints (**Table 1**) (26–28). Evidence from solid tumor models supports NGF/TrkA and TrkB.FL as drivers of proliferation and invasion through MAPK, PI3K–AKT and PLCγ signaling, while kinase-deficient splice variants such as TrkB.T1 modulate oncogenic pathways and may differentially affect communication with the OS immune microenvironment.

**Table 1:** Core features of NGF/TrkA and BDNF/TrkB signaling relevant to osteosarcoma biology and antitumor immunity.

Axis	Receptor features (OS-relevant)	Proximal signaling nodes	Dominant tumor-cell programs in OS models	Immune/stromal interfaces
NGF/TrkA	High-affinity NGF binding; kinase-active TrkA; co-modulation by p75^NTR^	ERK1/2, PI3K–AKT–mTOR, PLCγ	Motility and invasion via MMP-linked matrix remodeling; survival signaling under stress	Regulation of leukocyte adhesion/trafficking; potential effects on endothelial activation and perivascular remodeling
BDNF/TrkB	High-affinity BDNF binding; co-expression of kinase-active TrkB.FL and truncated TrkB.T1 isoforms in tumors	PI3K–AKT, ERK, PLCγ	Survival, anoikis resistance, invasion; pro-angiogenic VEGF induction	TrkB on dendritic cells suggests modulation of maturation and T-cell priming; potential impact on myeloid polarization

Within OS, patient-level transcriptomic and tissue analyses indicate that NGF is expressed at comparatively high levels and associates with prometastatic behavior. Functional studies show that NGF increases motility and invasiveness of OS cells through MMP-2–dependent matrix remodeling, consistent with a TrkA-coupled enhancement of migratory programs (29–31). BDNF/TrkB biology in OS, particularly the distribution of TrkB.FL and TrkB.T1 transcript variants, is less comprehensively mapped than NGF/TrkA, but convergent data from bone and soft-tissue contexts underscore relevant mechanisms. In chondrogenic and endothelial systems, BDNF/TrkB increases VEGF expression and augments angiogenesis through PI3K–AKT, a pathway with direct relevance to OS vascular remodeling in mineralized tissues (32–35). In epithelial and neuroendocrine tumors, TrkB promotes survival, invasion and resistance to detachment stress (anoikis) via ERK, PI3K–AKT and PLCγ; these conserved signaling solutions are detectable in OS models and likely contribute to adaptation within the hypoxic, stiff bone niche (36–38). These features support BDNF/TrkB as a candidate determinant of tumor persistence and dissemination in OS.

Neurotrophin signaling also interfaces with antitumor immunity in ways pertinent to OS. Human monocyte-derived dendritic cells express TrkB, and BDNF modulates their maturation, implying that BDNF/TrkB can alter antigen-presenting cell function and subsequent T-cell priming (39–41). NGF/TrkA has been linked to leukocyte adhesion and trafficking programs, suggesting that neurotrophin gradients can influence the composition and spatial organization of myeloid and lymphoid subsets in the tumor microenvironment (42–44). Given the myeloid-rich, T-cell-sparse landscape typical of OS, these receptor–ligand systems plausibly contribute to immune exclusion and dysfunctional activation states that blunt cytotoxic immunity.

Careful anatomical and temporal annotation of receptor signaling, together with explicit consideration of murine–human differences in NGF/BDNF expression, Trk isoforms, ligand affinity and immune-cell repertoires, is required to relate cell-intrinsic effects to stromal and immune phenotypes and to gauge the translational limits of murine OS models. Such standardization improves interpretability across studies.

## 3 Mechanisms of neurotrophin-driven tumor–immune remodeling

Neurotrophin signaling reprograms the osteosarcoma microenvironment through coordinated actions on myeloid trafficking and polarization, antigen-presenting cell function, endothelial activation, and neuron–immune circuits that collectively degrade antitumor effector quality. In the myeloid compartment, NGF–TrkA augments adhesion molecule programs that favor monocyte ingress and positioning (45–47). Experimental work demonstrates that NGF upregulates leukocyte adhesion pathways and facilitates monocyte–endothelium interactions while skewing macrophage phenotype, providing a plausible route to TAM accumulation and function in OS (48–50). Together with TRKA-dependent induction of IL-10 in human macrophages exposed to tumor-derived danger signals, these data support a model in which NGF promotes immune suppressive myeloid states that hinder cytotoxic lymphocyte activity within mineralized tumor beds. (**Table 2**).

**Table 2:** Mechanistic map of NGF/TrkA and BDNF/TrkB effects on immune and stromal components in osteosarcoma.

Mechanistic focus	Principal responding cells in OS milieu	Dominant signaling nodes engaged	Resulting immune/stromal outcome in OS
Monocyte recruitment and macrophage polarization under NGF–TrkA gradients	Circulating monocytes; tumor-associated macrophages	TrkA→PI3K–AKT; NF-κB/p38; adhesion modules (ICAM-1/VCAM-1)	Increased myeloid influx; enrichment of immunosuppressive TAM phenotypes; elevated IL-10; impaired cytotoxic T-cell activity
DC maturation under BDNF/TrkB	Conventional dendritic cells (monocyte-derived and tissue DCs)	TrkB→ERK/AKT; maturation program modulators	Altered costimulation and cytokine secretion; modified T-cell priming quality and breadth
Endothelial activation and vascular remodeling by NGF–TrkA	Endothelial cells; endothelial progenitors; perivascular stromal cells	TrkA→PI3K–AKT→MMP-2; ERK; pro-angiogenic factors (VEGF, FGF2)	Pro-angiogenic, tortuous vasculature; reduced T-cell trafficking; preferential myeloid recruitment
Neuron–immune coupling within innervated tumor bone	Sensory neurons; perivascular nerves; innate and adaptive immune cells	Neurotrophin-responsive neuronal pathways; neuropeptide release; secondary activation of immune checkpoints	Reinforcement of immune-suppressive tone (MDSC support, T-cell dysfunction); stabilization of immune-excluded architecture
Compartmental integration across the bone niche	Osteoblastic/osteoclastic stromal units; vasculature; myeloid and lymphoid subsets	Convergent ERK/AKT/PLCγ axes downstream of Trk receptors	Context-dependent dominance of myeloid programs with dampened effector T-cell infiltration and function

Dendritic cell (DC) interfaces with neurotrophins are also relevant to T-cell priming quality in OS. Human DCs express TrkB, and exogenous BDNF or NT-4 directly modulates their maturation, consistent with a capacity for TrkB ligands to alter costimulation and cytokine output during antigen presentation (51–53). Such shifts, together with BDNF/TrkB-driven epithelial-to-mesenchymal transition (EMT) reported in other tumors, would be expected to favor dysfunctional activation and immune evasion in a microenvironment already characterized by poor effector infiltration (54–56). While the magnitude and direction of these effects likely depend on local cytokine tone and receptor stoichiometry, the presence of a DC-intrinsic TrkB pathway provides a mechanistic substrate for neurotrophin-driven remodeling of adaptive responses in OS.

Endothelial and perivascular programs offer a second axis through which neurotrophins can reshape antitumor immunity. NGF–TrkA promotes endothelial invasion and tube formation through PI3K–AKT-dependent induction of MMP-2, and enhances endothelial progenitor recruitment and differentiation; both processes are coupled to VEGF and FGF2 induction and can generate aberrant, immunomodulatory vasculature (57–59). In OS, where endothelial activation and vascular patterning influence leukocyte entry and spatial organization, NGF-driven angiogenic signaling provides a credible mechanism for lymphocyte exclusion and myeloid-biased trafficking at tumor–bone interfaces.

Neuronal inputs further integrate with immune regulation in ways that intersect with neurotrophin biology. Sensory nociceptors, which are themselves shaped by neurotrophins, can diminish immune surveillance by fostering myeloid-derived suppressor cell programs and T-cell dysfunction in solid tumors. Related studies show that nociceptor–tumor interactions impair antitumor immunity and alter neurite outgrowth and mediator release, consistent with a feed-forward loop in which neurotrophin-dependent axonogenesis and neuronal activity reinforce immune suppression (60–62). These mechanisms are pertinent to OS, a disease with increasing evidence of tumor-infiltrating nerves and neuroimmune crosstalk, and underlie pain, functional limitation and quality-of-life impairment that should be captured as endpoints in future trials.

These cell-type–specific effects converge within the bone niche, where myeloid predominance, endothelial remodeling and stromal programs are positioned to respond to neurotrophin inputs, and emerging data suggest that NGF/BDNF and Trk expression can diverge between primary bone lesions and lung metastases, shaping site-specific immune escape and treatment response. Mapping ligand–receptor activity across bone and lung sites, including NGF/BDNF- or Trk-bearing extracellular vesicles that reprogram distant immune cells and pre-metastatic niches, should therefore support mechanistic attribution of vesicle-derived neurotrophin signals and development of fluid biomarkers.

## 4 Therapeutic strategies targeting NGF/TrkA and BDNF/TrkB

**Figure 1:**
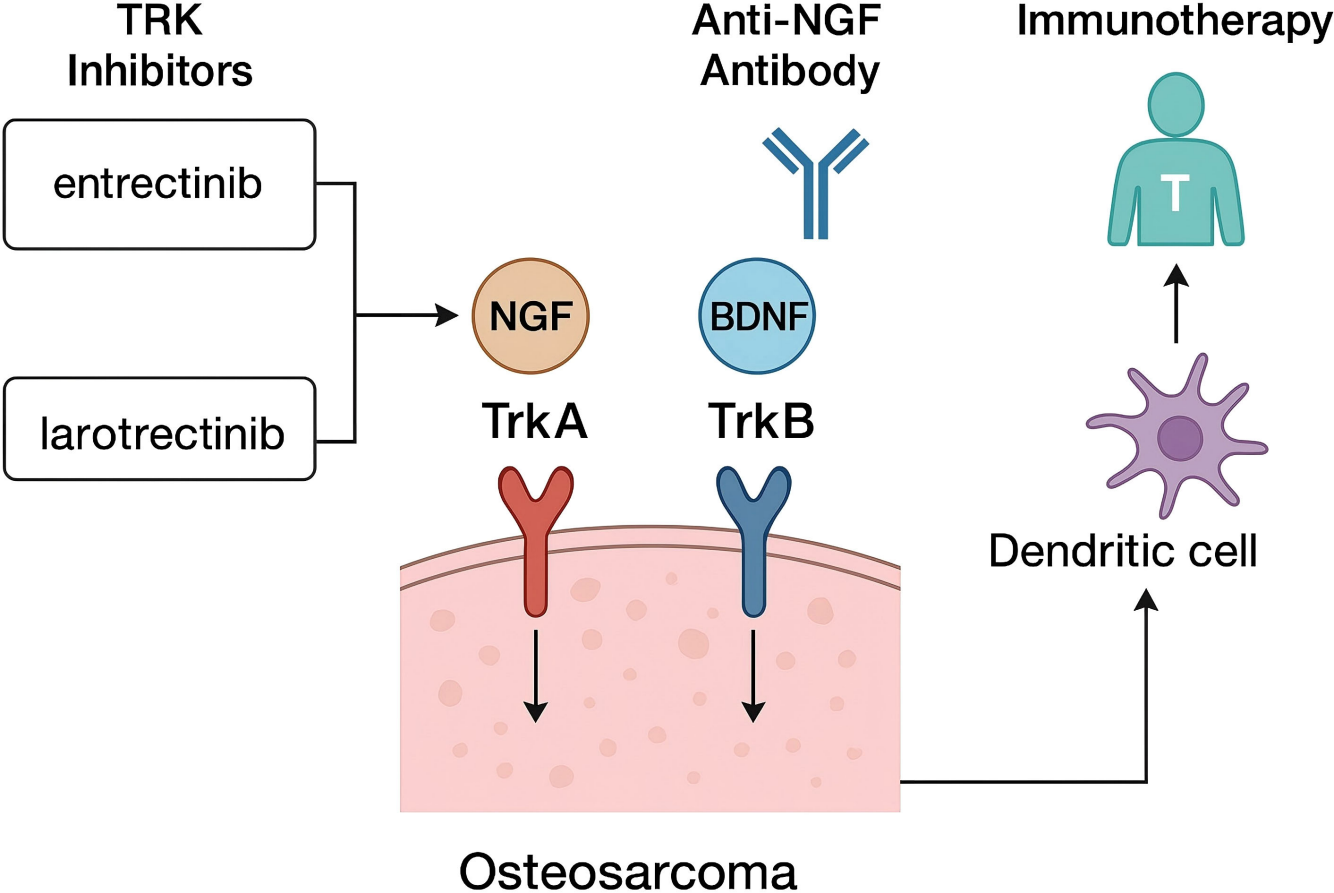
Therapeutic entry points in osteosarcoma: TRK inhibition (NGF/TrkA, BDNF/TrkB) and immunotherapy.

As shown in **Figure 1**, pharmacologic interruption of neurotrophin signaling in osteosarcoma can be approached at the receptor kinase level or at the ligand–receptor interface. Clinically available TRK tyrosine-kinase inhibitors (TRKis) provide the most immediate avenue but act only on kinase-active receptor isoforms. Entrectinib and larotrectinib produce high objective response rates across tumour types harboring NTRK fusions, with durable disease control; these approvals establish on-target druggability of Trk receptors and are relevant to osteosarcoma in the subset with actionable NTRK rearrangements (63–68). These studies support routine assessment for NTRK fusions in osteosarcoma and use of TRKis when present, with molecular re-profiling at progression to direct next-line TRKi selection.

Beyond canonical fusion-driven settings, preclinical work indicates that NGF–TrkA signaling can be pharmacologically suppressed to blunt prometastatic programmes even in fusion-negative osteosarcoma. In orthotopic models, larotrectinib inhibited NGF-induced lung metastasis and reduced MEK/ERK-dependent MMP-2 upregulation, aligning with patient and cell-based data showing NGF correlates with MMP-2 expression and promotes migration and invasion (69–71). Although these findings require clinical corroboration, they nominate TRK blockade as a strategy to disable NGF-driven stromal remodeling and dissemination in osteosarcoma irrespective of fusion status.

Neutralization of neurotrophin ligands represents a complementary concept with distinct risk–benefit considerations and could, in principle, target both kinase-active and truncated receptor variants such as TrkB.T1. Anti-NGF monoclonal antibodies have demonstrated analgesic activity in osteoarthritis but are associated with joint-specific safety signals, including increased rates of rapidly progressive osteoarthritis in phase III trials (72–74). While tumour-directed benefits have not been established, these agents illustrate the feasibility of systemic NGF sequestration in humans and, if repurposed for oncology, would warrant careful dose selection and musculoskeletal monitoring.

Translational integration with immuno-oncology will likely involve combining TRK or ligand-directed strategies with checkpoint inhibitors or myeloid-targeted agents, guided by the neurotrophin-dependent immune alterations described in osteosarcoma. TRKis have predictable neurological and metabolic adverse events related to pathway on-target effects; standardized mitigation strategies and predefined dose-adjustment schemas are available and should be embedded into combination trials (75–77). Study designs should also incorporate prospective biospecimen collection to map changes in myeloid composition, dendritic-cell maturation and vascular cues under NGF/TrkA or BDNF/TrkB blockade, and explore dendritic cell–based vaccines that leverage Trk expression on antigen-presenting cells as complementary immunotherapy strategies.

Clinical implementation should follow a molecularly triaged algorithm. Patients with NTRK fusions should receive larotrectinib or entrectinib with serial ctDNA or tissue sequencing at progression to identify resistance mutations guiding selitrectinib-based salvage. In the broader population without fusions, early-phase studies are justified to test TRKis as stromal- and immunity-modulating agents in biomarker-enriched cohorts defined by NGF/TrkA or BDNF/TrkB activity (transcriptomic receptor–ligand scores, phospho-Trk IHC, or NGF-MMP-2 axis readouts). Trials should pre-specify immune endpoints (e.g., intratumoral CD8^+ T-cell density, dendritic-cell activation markers, and myeloid suppression indices) alongside conventional efficacy measures to quantify neurotrophin-directed immune recalibration. Safety monitoring ought to use harmonized toxicity definitions and structured reporting to capture both acute and delayed events, consistent with current immunotherapy pharmacovigilance practices.

## 5 Conclusions and future priorities

The collective evidence positions NGF/TrkA and BDNF/TrkB as context-dependent organizers of osteosarcoma biology and its immune microenvironment. Canonical Trk signaling through ERK, PI3K–AKT–mTOR and PLCγ integrates with mesenchymal lineage programs and stromal constraints to support survival, motility and matrix remodeling, while simultaneously influencing leukocyte trafficking and activation states (78–80). In parallel, BDNF/TrkB promotes angiogenic and anoikis-resistant phenotypes with plausible consequences for vascular patterning and immune cell entry (81–83). Neuroimmune coupling via sensory pathways further reinforces myeloid predominance and T-cell dysfunction in bone tumors, suggesting that neurotrophin gradients contribute to immune exclusion and impaired effector quality (84–86). These observations justify systematic evaluation of neurotrophin axes as therapeutic and biomarker targets in osteosarcoma.

Translational opportunities are immediate in genomically defined subsets and plausible in fusion-negative disease. TRK tyrosine-kinase inhibitors have established clinical activity across NTRK fusion-positive malignancies and should be integrated wherever fusions are identified, with molecular profiling at progression to guide next-generation TRKi selection (87–89). In orthotopic osteosarcoma models lacking fusions, pharmacologic TrkA blockade dampened NGF-induced MMP-2 programs and reduced metastatic spread, consistent with the NGF/MMP axis observed in patient and cell-based datasets (90, 91). Ligand-directed strategies are conceptually attractive but require careful risk assessment: anti-NGF monoclonal antibodies demonstrate on-mechanism analgesia yet carry joint-specific toxicities that are relevant to sarcoma populations with musculoskeletal comorbidities (92–94). Trial designs that combine TRK pathway inhibition with immunotherapy should prespecify immune endpoints and embed dose-modification schemas that reflect known on-target neurological and metabolic adverse events.

Future work should prioritize a standardized biomarker framework that resolves cell-type, spatial and temporal features of Trk signaling in human osteosarcoma and supports sex-stratified, puberty-aware analyses of neurotrophin, Trk and immune pathways. Practical components include phospho-Trk immunohistochemistry and multiplex spatial profiling to map signaling to myeloid infiltration, dendritic-cell maturation and endothelial activation; transcriptomic receptor–ligand activity scores to stratify patients; and functional readouts such as NGF–MMP-2 axis activity and VEGF-linked angiogenic signatures (95–98). Harmonized definitions for immunologic outcomes—intratumoral CD8^+ T-cell density, dendritic-cell activation markers and myeloid suppression indices—together with uniform safety reporting will enable comparability across studies and accelerate iteration of severity-based management algorithms, an approach that has improved clinical translation in adjacent cellular-therapy fields.

Clinical testing should follow a molecularly triaged algorithm anchored in reproducible assays. Patients with NTRK fusions should receive approved TRK inhibitors with serial genomic reassessment to identify resistance mutations and rationally sequence next-generation agents. For fusion-negative disease, early-phase trials should enrich for high NGF/TrkA or BDNF/TrkB activity using multi-parameter criteria and incorporate correlative studies that quantify changes in myeloid composition, dendritic-cell maturation and vascular cues under pathway blockade (99–101). Because nociceptor–tumor signaling intersects with neurotrophin biology, prospective capture of pain and neurosensory parameters is warranted, while monitoring for joint events if anti-NGF strategies are explored (102–105). Given the potential for vascular and adhesion-program modulation to alter leukocyte entry, trials should integrate imaging or biopsy-based assessments of endothelial activation to relate vascular remodeling to T-cell trafficking.

NGF/TrkA and BDNF/TrkB constitute actionable determinants of osteosarcoma progression and antitumor immunity. Mechanistic data across tumor-cell, endothelial, neuronal and myeloid compartments support therapeutic interrogation of these axes, with fusion-directed TRK inhibition as standard of care where applicable and biomarker-guided investigation of pathway blockade in the broader population. Methodologically rigorous studies that couple pathway inhibition to predefined immune and clinical endpoints, deploy standardized biospecimen workflows and use harmonized safety reporting are required to determine whether neurotrophin-targeted strategies can remodel the osteosarcoma microenvironment toward durable immune control.
